# Transactions of the Pathological Society of Philadelphia

**Published:** 1840-04-11

**Authors:** 


### MEDICAL EXAMINER.

DEVOTED TO MEDICINE, SURGERY, AND THE COLLATERAL SCIENCES.

No. 15.] PHILADELPHIA, SATURDAY, APRIL 11, 1840. [Vol. III.

### TRANSACTIONS OF THE PATHOLOGICAL SOCIETY OF PHILADELPHIA.

*April*, 1840.

The President, Dr. Gerhard, in the Chair.

*Tumour of Left Labium, resembling Elephantiasis, removed from a negress.* By Isaac Parrish, M. D.

Eliza Collins, a negress, aged 19 years, of robust frame and healthy constitution, first came under my notice last autumn.

I was requested to visit her by my friend, Dr. Gregg, whom she had consulted on account of a tumour of the left labium pudendi. On examination, a large tumour of an oblong form, was found hanging between the thighs and extending almost to the knees; its base occupied the whole of the labium, being moveable and loosely attached. The skin covering the upper third of the tumour on its outer side, was smooth and perfectly natural; in the other parts it was rough and corrugated, resembling, very closely, the appearance observed in elephantiasis. On the inner and upper portion, a sort of sulcus was formed by a portion of the vagina being drawn down. This part preserved the mucous character of the vagina, and was extremely sensitive to the touch; the urine passed over this vaginal portion, producing, at times, excoriation and pain. There was no pain in the body of the tumour; it was of a dense, elastic structure, being equally compressible over its whole surface. The parts within the vagina appeared to maintain their natural position and functions.

The patient was at this time, far advanced in pregnancy, and it was not considered prudent to attempt a removal of the tumour, especially as it could not interfere with the passage of the child's head. In this opinion Dr. Hodge concurred, having examined the patient at our request. The operation was therefore deferred until after parturition. The patient gave the following history of her case. She was married at the age of 15, at which time she was perfectly healthy. Soon after marriage, she discovered a small lump on the side of the labium towards the groin; this increased gradually during pregnancy, and at the time of the birth of her first child, about a year after marriage, it was the size of a large orange. It was slightly painful, but did not prevent her from moving about.

She was delivered in due time, but the child was still-born. The tumour continued to increase, until she again became pregnant, when it grew more rapidly, and at the time of our examination, it had attained a size somewhat larger than at present. The patient gave birth to her second child last christmas. The infant, as she supposes, was of full size, but died in twenty-four hours after birth. Nothing unusual occurred during the labour that the patient is aware of. After parturition, the tumour decreased in size for several weeks, after which it began to grow again, until it reached its present dimensions.

On the morning of the 18th inst., I removed the tumour by making an elliptical incision around its base, and dissecting it off from its connections. It was necessary to divide that portion of the vagina which had been drawn down, and which occupied its inner and upper surface.

But two arteries required ligatures; there was, however, copious hæmorrhage from several large veins which entered the tumour at its base, and it was necessary to secure two of these, before the bleeding ceased. The parts were brought together by sutures, and the requisite dressings applied.

The patient is doing well at this date.

Drs. Barton, Ashmead, Kirkbride, Gregg, Warrington, and several medical students were present at the operation.

In cutting into the tumour after its removal, it was found to consist of a white, opaque substance, of uniform density and structure, without any appearance of distinct fibres or of sacs. A thin serous fluid issued from the cut surface in considerable quantity; the orifices of several venous trunks were seen, which could be traced by the probe through the body of the tumour, terminating under the skin in its most depending portion. The substance of this curious growth appeared to us to be condensed cellular tissue, while the appearance of the skin which covered it, bears a close resemblance to elephantiasis. It occurred to us that it might be nothing more than an elephantiasis of the labium, which had assumed this particular form, and manner of growth, from the peculiar structure and position of the part from which it originated. This, however, is a mere suggestion, to test the correctness of which, a more minute examination will be required.

The dimensions of the tumour are as follows: horizontal circumference, one foot four inches; greatest diameter seven inches; greatest diameter of base four inches, least diameter two inches. Its weight was two pounds, eight ounces, avoirdupois.

